# Toward more efficient ergothioneine production using the fungal ergothioneine biosynthetic pathway

**DOI:** 10.1186/s12934-022-01807-3

**Published:** 2022-05-07

**Authors:** Zhihui Chen, Yongzhi He, Xinyu Wu, Li Wang, Zhiyang Dong, Xiuzhen Chen

**Affiliations:** 1 State Key Laboratory of Microbial Resources, Institute of Microbiology, Chinese Academy of Sciences, Beijing, 100101 China; 2 University of Chinese Academy of Sciences, Beijing, 100049 China

**Keywords:** Ergothioneine, Biosynthesis, Heterologous expression, *Trichoderma reesei*, *Neurospora crassa*, *Escherichia coli*

## Abstract

**Background:**

Ergothioneine (ERG) is a potent histidine-derived antioxidant that confers health-promoting effects. Only certain bacteria and fungi can biosynthesize ERG, but the ERG productivity in natural producers is low. ERG overproduction through genetic engineering represents an efficient and cost-effective manufacturing strategy.

**Results:**

Here, we showed that *Trichoderma reesei* can synthesize ERG during conidiogenesis and hyphal growth. Co-expression of two ERG biosynthesis genes (*tregt*1 and *tregt*2) from *T. reesei* enabled *E. coli* to generate 70.59 mg/L ERG at the shaking flask level after 48 h of whole-cell biocatalysis, whereas minor amounts of ERG were synthesized by the recombinant *E. coli* strain bearing only the *tregt*1 gene. By fed-batch fermentation, the extracellular ERG production reached 4.34 g/L after 143 h of cultivation in a 2-L jar fermenter, which is the highest level of ERG production reported thus far. Similarly, ERG synthesis also occurred in the *E. coli* strain engineered with the two well-characterized genes from *N. crassa* and the ERG productivity was up to 4.22 g/L after 143 h of cultivation under the above-mentioned conditions.

**Conclusions:**

Our results showed that the overproduction of ERG in *E. coli* could be achieved through two-enzymatic steps, demonstrating high efficiency of the fungal ERG biosynthetic pathway. Meanwhile, this work offers a more promising approach for the industrial production of ERG.

**Supplementary Information:**

The online version contains supplementary material available at 10.1186/s12934-022-01807-3.

## Background

Ergothioneine (ERG) is a thiol-containing histidine betaine derivative, that protects cells against oxidative damage caused by excess reactive oxygen species (ROS). Uniquely, ERG exists predominately in the thione tautomer at physiological pH [1] and possesses relatively high reduction potential (−60 mV), making it more stable and resistant to autooxidation than other thiol-containing antioxidants such as glutathione [2]. As a powerful antioxidant and cytoprotectant, ERG has been suggested to confer effective and beneficial roles on human health, such as anti-inflammation [3], anti-ageing [4], and antidepressant properties and the ability to prevent ultraviolet damage [5–7]. Recently, ERG has been assessed as safe to use in the food and cosmetics industries [8–10], which will increase the market demand for ERG and exploration of methods to produce ERG [11].

ERG biosynthesis occurs only in certain bacteria and fungi, typically actinobacteria [12], cyanobacteria [13], methylobacteria [14], and various fungi including *Neurospora crassa* [15], the fission yeast *Schizosaccharomyces pombe* [16] and basidiomycetes mushrooms [17], but not in plants and animals. Cultivation or fermentation of natural ERG producers such as mushrooms, is an important commercial ERG production method. However, this method, which relies heavily on natural producers, has inherent deficiencies, such as low productivity and a long culture period [18], which leads to a limited ERG supply and high production costs. As such, an alternative and cost-effective approach to produce ERG is desired. With the characterization of ERG biosynthetic pathways in bacteria [19, 20] and fungi [15, 16], more interest has shifted to the fermentation production strategy using a microorganism overexpressing ERG biosynthetic genes. For non-ERG producers, bioproduction of ERG has been achieved through introducing the ERG biosynthetic gene cluster from *Mycobacterium smegmatis* into *E. coli* [21]*,* engineering *S. cerevisiae* with the *Grifola frondosa egt*1/*egt*2 genes [22] or with the combined use of *N. crassa egt*1 *and Claviceps purpurea egt*2 [23]. Of note, high production of ERG (1.31 g/L) was achieved in *E. coli* after 216 h in a 3-L jar fermenter by expressing five ERG biosynthetic genes (*egt*ABCDE) from *M. smegmatis,* enhancing l-cysteine (l-Cys) production, knocking out *met*J and optimizing the fermentation medium [24]. Likewise, recombinant expression of ERG biosynthetic genes has substantially increased the ERG productivity of natural ERG producers such as *S. pombe* [16] and *Aspergillus oryzae* [25]. Therefore, this approach represents an efficient and cost-effective means for the industrial production of ERG [21]. However, the output of ERG is still relatively low, resulting in insufficient supply of ERG and high price. Sequence-based phylogenies of the key genes (*egt*B, *egt*D in *M. smegmatis* and *egt*1 in *N. crassa*) revealed that there are far more bacterial species and fungal phyla capable of producing ERG than the number of ERG-producing microorganisms discovered thus far [12]. Exploring the potential of these microorganisms may be the key toward high-level ERG production, particularly for the fungal ERG biosynthetic pathway represented by *N. crassa* that requires only two genes and may be more effective in ERG biosynthesis. Until now, only a few fungal ERG biosynthesis genes have been characterized, and in terms of the ERG productivity, their potential does not seem to be fully realized in the fungal systems reported previously [22, 25]. Hence, it will be a valuable attempt to investigate the efficiency of various fungal ERG biosynthesis genes of synthesizing ERG in bacterial systems like *E. coli* that is a model microorganism commonly used for synthetic biology and industrial applications.

The filamentous fungus *Trichoderma reesei* is the workhorse for the industrial production of lignocellulolytic enzymes [26, 27]. Moreover, *T. reesei* is an attractive host for the production of recombinant proteins due to its extraordinary ability to secrete proteins and its (GRAS) Generally Regarded as Safe status approved by the US Food and Drug Administration [28–30]. Although the physiological roles of ERG in *T. reesei* remain unknown, the presence of putative ERG biosynthetic genes raises the possibility that *T. reesei* has the potential to produce ERG.

Here, we determined that *T. reesei* can synthesize ERG. Through heterologous expression in *E. coli*, we examined the role of the two putative ERG biosynthesis genes from *T. reesei* and investigated the possibility of synthesizing ERG in *E. coli* using the fungal ERG biosynthetic genes from *T. reesei* and *N. crassa*. Our research showed that high-level of ERG production in *E. coli* can be achieved by using only two genes from fungi. This work offers a more practical and promising approach for the industrial production of ERG.

## Results and discussion

### Cloning of ERG biosynthesis genes from T. reesei

**Fig. 1:**
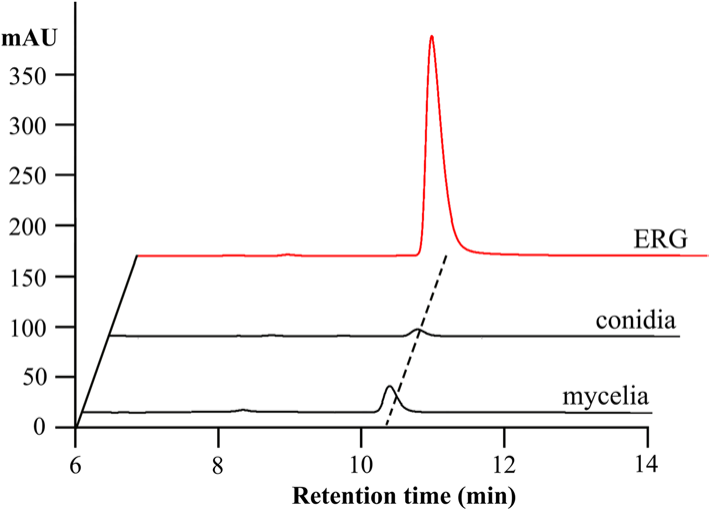
HPLC analysis of ERG content of *T. reesei* conidia and mycelia. “ERG” indicates the ERG standards (50 mg/L)

Prior to cloning the ERG biosynthesis genes, we extracted ERG from the conidia and mycelia to determine whether *T. reesei* has evolved the ability to synthesize ERG. HPLC analysis showed that the extracted samples displayed a predominant peak at a retention time of 10–10.5 min, which was the same as that of the ERG standard (Fig. 1). The predominant peak was further confirmed by LC–MS analysis (Additional file 1: Fig. S1). These results clearly demonstrated that *T. reesei* can synthesize ERG*,* which provides a basis for cloning of functional ERG biosynthetic genes in *T. reesei*.

**Fig. 2:**
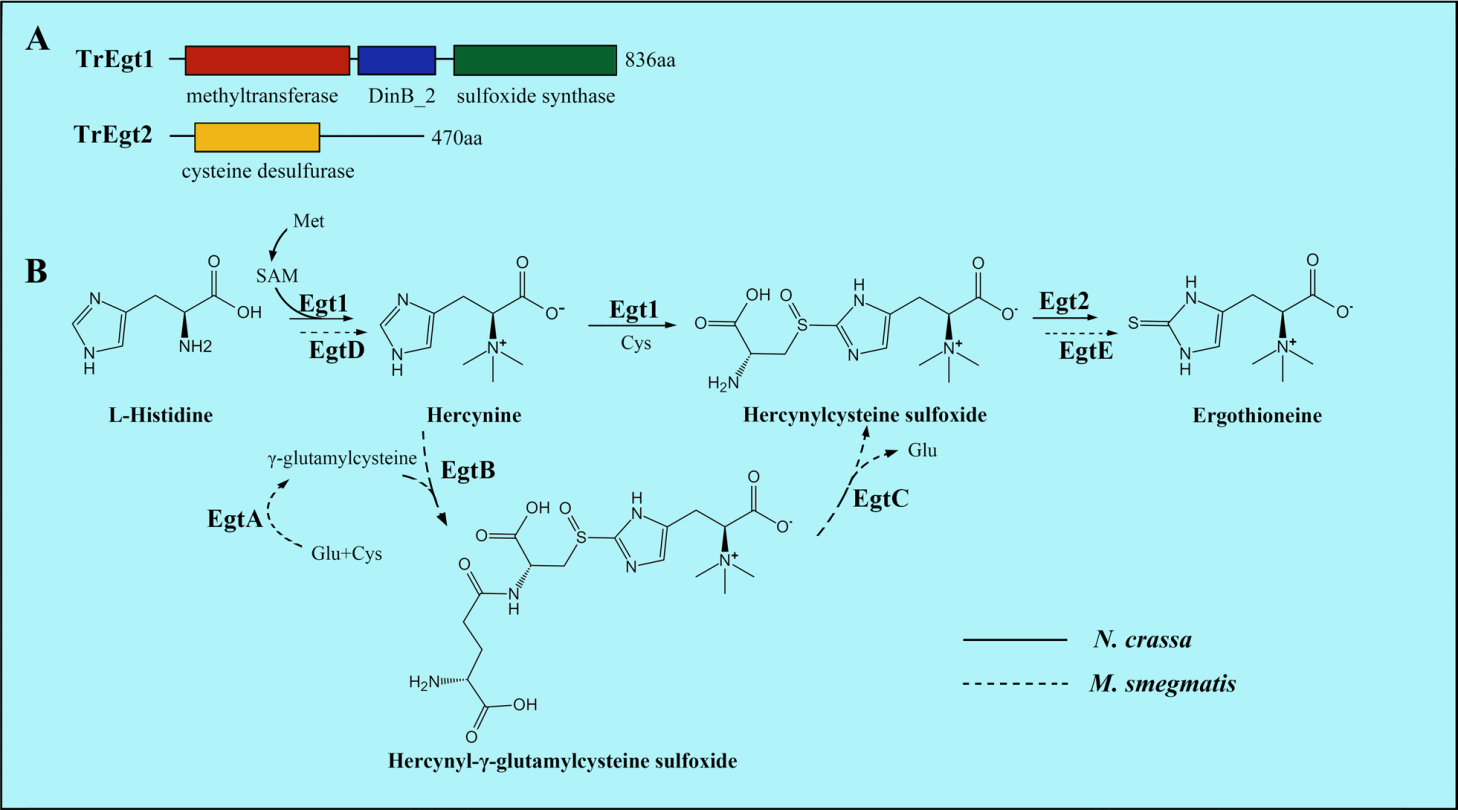
The biosynthetic pathway of ergothioneine (ERG) and the domain prediction of Tregt1 and Tregt2. **A** Conserved domain predictions of Tregt1 (NCBI Reference Sequence: XP_006968620) and Tregt2 (NCBI Reference Sequence: XP_006968735) from *T. reesei*. **B** The biosynthetic pathway of ERG in *Neurospora crassa* (solid arrow) and *Mycobacterium smegmatis* (dashed arrow). The ERG biosynthesis in *N. crassa* requires only two main enzymes (Egt1 and Egt2) while in *M. smegmatis*, a gene cluster egtABCDE is responsible for ERG biosynthesis. Egt1 is a bifunctional enzyme that catalyzes the first two steps: the addition of three methyl groups in l-histidine to form hercynine, and the formation of C–S bond between l-cysteine (l-Cys) and hercynine to form hercynylcysteine sulfoxide. *M. smegmatis* sulfoxide synthase utilizes γ-glutamylcysteine (γGC) but not cysteine as sulfur donor. l-Met, l-methionine; SAM, S-adenosylmethionine; l-Cys, l-cysteine; l-Glu, l-glutamic acid

BLASTP search with *N. crassa* Ncegt1 ((NCBI Reference Sequence: XP_956324) and Ncegt2 (NCBI Reference Sequence: XP_001728131) as query sequences revealed that two hypothetical proteins designated Tregt1 (NCBI Reference Sequence: XP_006968620) and Tregt2 (NCBI Reference Sequence: XP_006968735), respectively, were probably involved in ERG biosynthesis in *T. reesei*. The coding sequences of the cloned *tregt*1 and *tregt*2 genes were 2502 bp and 1413 bp (Additional file 1: Data S1, S2), respectively. Tregt1 (Additional file 1: Data S3) shared 61.28% (97% coverage) amino acid sequence identity with Ncegt1 and contained an S-adenosylmethionine (SAM)-dependent methyltransferase domain, a DinB_2 domain, and a sulfoxide synthase domain (Fig. 2A), implying that Tregt1 may catalyze the first two steps of the ERG biosynthetic pathway.

Tregt2 (Additional file 1: Data S4), a putative selenocysteine lyase-like protein, displayed 53.77% homology (98% coverage) with NcEgt2 and included the pyridoxal phosphate (PLP)-dependent cysteine desulfurase domain present in Ncegt2 that catalyzes the conversion of hercynylcysteine sulfoxide to ergothioneine by cleaving the C–S bond. It is probably that like *N. crassa* [15]*, T. reesei* synthesizes ERG through two enzymes instead of five-enzymatic catalysis for ERG biosynthesis in *M. smegmatis* [19] (Fig. 2B).

### ERG biosynthesis by a whole-cell biocatalyst

**Fig. 3:**
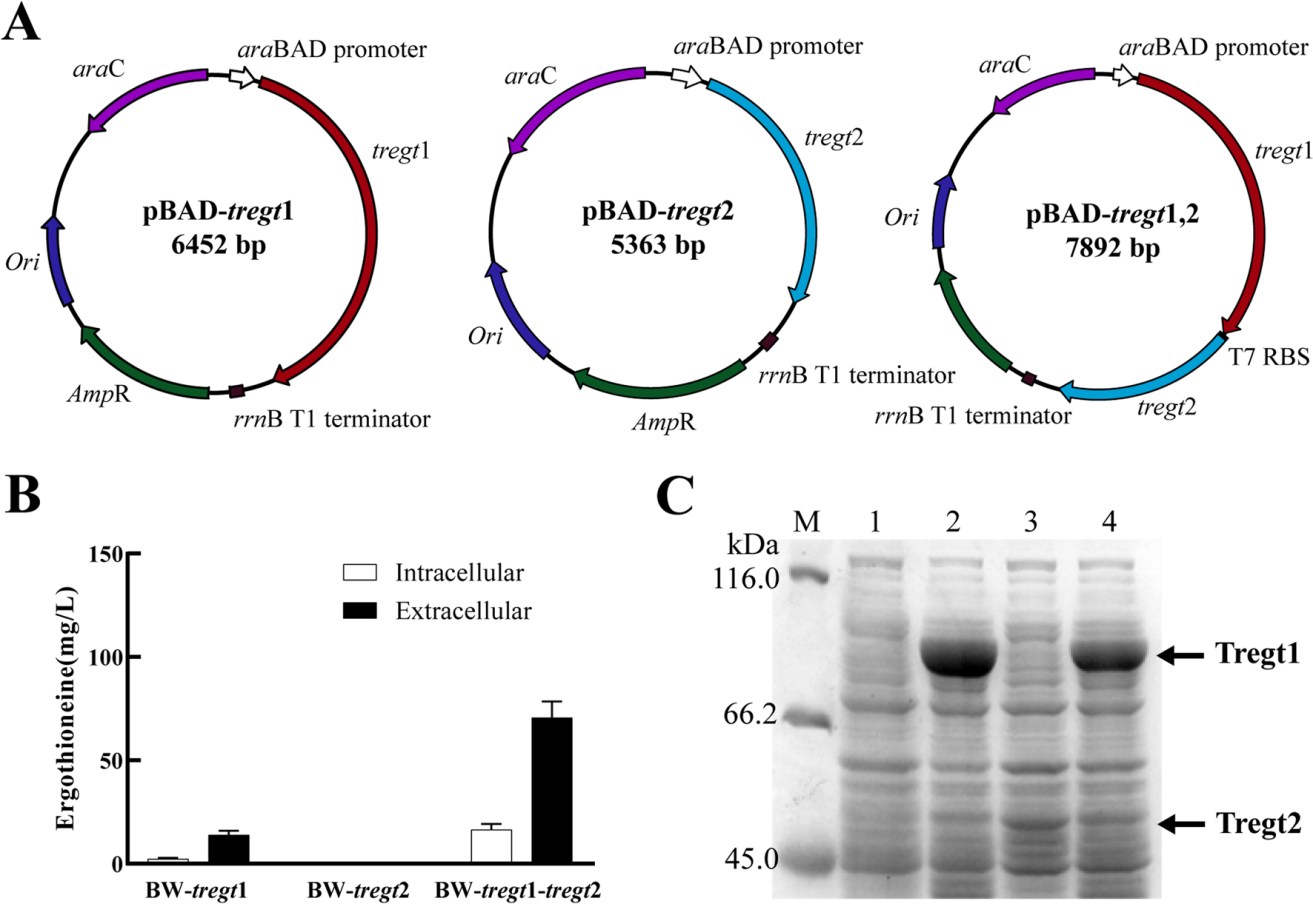
Plasmid profiles, protein expression and ERG biosynthesis. **A** Schematic drawing of plasmids expressing *tregt*1 and/or *tregt*2 used for transforming *E. coil* BW25113. **B** The production of ERG by 48-h whole cell catalysis using the recombinant strains BW-*tregt*1, BW-*tregt*2 and BW-*tregt*1-*tregt*2. Data in the figure are mean values (n = 3 biological replicates). **C** Detection of Tregt1 and/or Tregt2 expression in recombinant *E. coli* by SDS-PAGE. M. Protein marker; 1. BW-pBAD (control); 2. BW-*tregt*1; 3. BW-*tregt*2; 4. BW-*tregt*1-*tregt*2

To test whether Tregt1 and Tregt2 have the ability to convert l-histidine into ERG, we constructed recombinant *E. coli* strains harbouring the expression plasmids pBAD, pBAD-*tregt*1, pBAD-*tregt*2, and pBAD-*tregt*1-*tregt*2(Fig. 3A). After 48 h of whole-cell biocatalyst reaction, the strain bearing pBAD-*tregt*1-*tregt*2 produced 70.59 mg/L extracellular ERG. However, ERG was not detected from the strain with pBAD-*tregt*2 (Fig. 3B), although *tregt*2 was successfully expressed in this recombinant *E. coli* strain (Fig. 3C). Of note, the strain with pBAD-*tregt*1 was able to synthesize ERG, although its production was lower than that of recombinant strain co-expressing *tregt*1 and *tregt*2, suggesting that functions of Tregt2 may be performed by other yet unknown enzymes with weak cleavage activity of hercynylcysteine sulfoxide, which was also observed in *Saccharomyces cerevisiae* [22].

To test whether ERG biosynthesis in *E. coli* can also be achieved by using two genes from other fungi, we constructed the recombinant *E. coli* strain bearing *egt*1 and *egt*2 genes from *N. crassa.* Similarly, we found that the co-expression of *egt*1 and *egt*2 from *N. crassa* also enabled *E. coli* to produce ERG (Additional file 1: Fig.S2), showing that it is practical to synthesize ERG in *E. coli* only using two genes originating from fungi.

### ERG production by high-cell-density fermentation

**Fig. 4:**
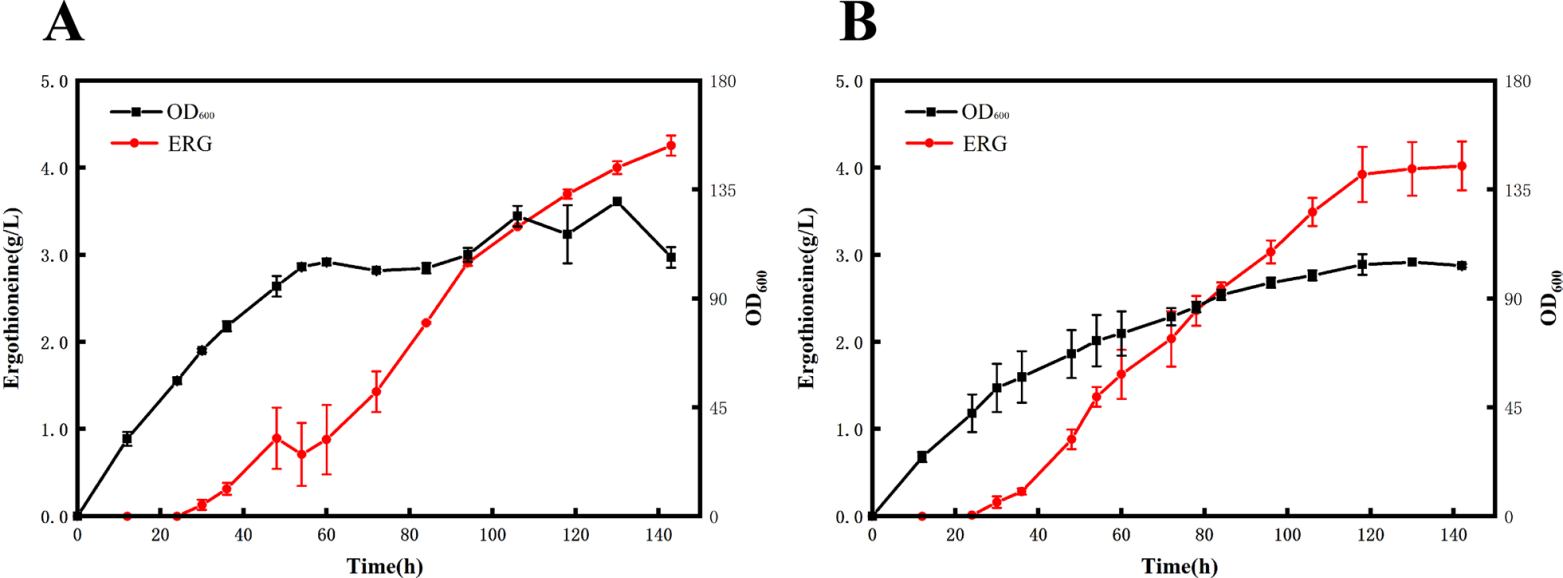
ERG production of the recombinant strains in a 2-L jar fermenter. The production of ERG using the recombinant strains BW-*tregt*1-*tregt*2 **A** and BW-*ncegt*1-*ncegt*2 **B** during high-cell-density fermentation. Data in the figure are mean values (n = 3 biological replicates)

To evaluate the potential of the recombinant *E. coli* strain co-expressing *tregt*1 and *tregt*2 for the industrial production of ERG, we performed high**-**cell**-**density fermentation in a 2**-**L jar fermenter with the fed**-**batch strategy. During the whole fermentation process, the recombinant strain grew well, and the OD_600_ of the cultures reached 105 at 60 h and 130 at 130 h. Extracellular ERG was detected at 30 h and continued to increase until 143 h, with the concentrations of ERG in the supernatant of 0.89 g/L at 48 h, 1.43 g/L at 72 h, 2.91 g/L at 94 h and 4.34 g/L at 143 h (Fig. 4A), which is the highest ERG production level reported thus far. Similarly, high level of ERG production (4.22 g/L) was achieved in the *E. coli* strain bearing the two genes responsible for *N. crassa* ERG biosynthesis (Fig. 4B). In addition to the contribution of the fermentation conditions to ERG production, another important reason for the high yield of ERG is probably due to the utilization of the genes associated with the fungal ERG biosynthesis. Compared to that from *M. smegmatis*, the fungal ERG biosynthetic pathway represented by *N. crassa* is more effective since it requires only two genes and l-Cys rather than γ-glutamylcysteine (γGC) as a sulfur donor, which facilitates hercynylcysteine sulfoxide synthesis and avoids competition with the glutathione synthesis pathway [15, 19]. From our results, the effectiveness of the fungal ERG synthesis pathway can be achieved not only in fungi but also in bacteria; therefore, it is practical to overproduce ERG through heterologous expression of ERG biosynthetic genes from fungi in *E. coli*. To maximize the ERG productivity of the recombinant strain, we will conduct closer inspection of the gene expression level, intermediate product accumulation, ERG precursor supply and proportion, and further optimizations will be made.

## Conclusions

Here, we demonstrated that *T. reesei* can synthesize ERG. By bioinformatics analysis and reconstruction of the *T. reesei* ERG synthetic pathway in *E. coli,* we found that ERG biosynthesis in *E. coli* can be achieved by using only two genes from *T. reesei*, with Tregt1 being the key enzyme in this process. In addition, the recombinant *E. coli* strain co-expressing *egt*1 and *egt*2 genes from *N. crassa* also can synthesize ERG*.* Through fed-batch cultivation, the highest level of ERG production was achieved after 143 h of cultivation. To the best of our knowledge, this is the first report to overproduce ERG in *E. coli* with fungal biosynthetic genes.

## Materials and methods

### Strains and media

*Trichoderma reesei* strain QM9414 (ATCC 26,921) was cultivated on potato dextrose agar (PDA) or in liquid minimal medium (MM) with 5 g/L glucose and 40 g/L lactose as the carbon source. MM without peptone was prepared as described previously [31]. *E. coli* strain Trans1-T1 (TransGen Biotech, China) was used for standard cloning. *E. coli* K12/BW25113 (*rrn*B3 Δ*lac*Z4787 *hsd*R514Δ(*ara*BAD)567 Δ(*rha*BAD)568 *rph*-1) [32] was used as the host strain for the heterologous expression of ERG biosynthetic genes from *T. reesei* and ERG production. Luria–Bertani (LB) medium (10 g/L tryptone, 5 g/L yeast extract, 10 g/L NaCl) and ZYM auto-induction medium [33] was used to grow *E. coli* cells and to express enzyme, respectively. A defined medium (DM) was used for whole-cell biocatalysis and fed-batch fermentation, which contained (per L) 10 g of glucose, 8 g of (NH_4_)_2_HPO_4_, 13.3 g of KH_2_PO_4_, 1.2 g of MgSO_4_·7H_2_O, 1.7 g of citric acid and 10 mL of a trace metal solution. The trace metal solution (per litre of 5 M HCl) consisted of 10 g of FeSO_4_·7H_2_O, 2.25 g of ZnSO_4_·7H_2_O, 1 g of CuSO_4_·5H_2_O, 0.5 g of MnSO_4_·5H_2_O, 0.23 g of Na_2_B_4_O_7_·10H_2_O, 2 g of CaCl_2_·2H_2_O and 0.1 g of (NH_4_)_6_Mo_7_O_24_. When necessary, the antibiotic ampicillin (100 mg/L)or the inducer L-arabinose (2 g/L) was added.

### Extraction of ERG from T. reesei

*Trichoderma reesei* QM9414 was cultivated on PDA at 28 ℃ for 10 days, and then the conidia were suspended in 1.1 M sorbitol solution and centrifuged at 13,300 rpm for 3 min. The lower conidia pellet was frozen in liquid nitrogen and ground into a powder. After that, the conidial powder was added to an 85% methanol solution and vortexed for 1 min, and the suspension was centrifuged at 13,300 rpm for 3 min. The supernatant was collected and diluted tenfold with 70% acetonitrile solution, and then filtered through a 0.22 μm filter for ERG detection.

The conidia of *T. reesei* QM9414 were inoculated into liquid MM containing 0.5% (w/v) glucose and 4% (w/v) lactose and cultivated on a rotary shaker (200 rpm) at 28 ℃ for 6 days, and the mycelia were collected to extract ERG. The method of extracting ERG from mycelium was the same as that for the conidia.

### Construction of the recombinant BW25113 strains

The expression vectors were constructed with the plasmid pBAD/His (Invitrogen, USA) as the backbone, which includes the pBR322 origin, *ara*BAD promoter induced by arabinose and *rrn*B terminator. Primer pairs FpBAD/RpBAD were designed to amplify plasmid pBAD/His to obtain linearized pBAD/His for subsequent vector construction.

The entire open reading frame of the *tregt*1 and *tregt*2 genes were amplified using the primer pairs Ftregt1/Rtregt1 or Ftregt2/Rtregt2 with *T. reesei* QM9414 cDNA [34] as a template. The amplified *tregt*1 or *tregt*2 products were ligated with linearized pBAD/His through a Clone Express^®^ MultiS One Step Cloning Kit (Vazyme Biotech Co, China) and then transformed into *E. coli* Trans1-T1 for cloning and sequencing. The resulting plasmids pBAD-*tregt*1 and pBAD-*tregt*2, in which *tregt*1 or *tregt*2 was under the control of the *ara*BAD promoter and *rrn*B terminator, was used to transform BW25113 to obtain the recombinant strains BW-*tregt*1 and BW-*tregt*2. To construct the recombined *E. coli* strain co-expressing *tregt*1 and *tregt*2, the T7 RBS sequence (tgtttaactttaagaaggagatatacc) was used to link the two genes. Briefly, primer pairs Ftregt1/RpBAD-tregt1 were designed to amplify the plasmid pBAD-*tregt*1 to obtain linearized pBAD-*tregt*1 with the T7 RBS sequence attached to the 3′ end of *tregt*1. Additionally, *tregt*2 with the T7 RBS sequence at its 5′ end was amplified by PCR using the plasmid pBAD-*tregt*2 as template and the primer pairs FpBAD-tregt2/Rtregt2. Subsequently, plasmid pBAD-*tregt*1-*tregt*2 was constructed by ligating T7 RBS-containing *tregt*2 to pBAD-*tregt*1 harbouring T7 RBS, which was transformed into BW25113 to create the recombinant strain BW-*tregt*1-*tregt*2.

We adopted the same strategy to construct the expression plasmid pBAD-*ncegt*1-*ncegt*2 harbouring *egt*1 (XM_951231) and *egt*2 (XM_001728079.2)responsible for ERG biosynthesis in *N. crassa*, except that the entire open reading frames of the two genes were synthesized by Tsingke Biotechnology Co., Ltd (China). The resulting plasmid (Additional file 1: Fig.S2A) was transformed into BW25113 to obtain recombinant *E. coli* strain BW-*ncegt*1-*ncegt*2, in which *ncegt*1 and *ncegt*2 were also successfully expressed (Additional file 1: Fig.S2C).

All primers used in this study are listed in Table S1 in additional file 1.

### Protein expression and identification

BW25113 and the engineered strains derived from it were cultivated in LB medium with ampicillin at 37 ℃ on a rotary shaker (200 rpm) until the optical density of the cultures at 600 nm reached 0.6. Expression was induced by the addition of arabinose at a final concentration of 0.2% (w/v). After 24 h of induction at 30 ℃ and 200 rpm, the cells were collected by centrifugation and resuspended in 50 mM potassium phosphate buffer (pH 7.0). The cell suspension was sonicated and centrifuged (12,000×*g*, 10 min). The supernatant was used for SDS-PAGE analysis.

### Whole-cell catalysis conditions

BW25113 and the engineered strains derived from it were grown in LB medium overnight at 37 ℃ on a rotary shaker (200 rpm). Five hundred microliter of the overnight cultures were inoculated into 50 mL of ZYM auto-induction medium containing 2 g/L of arabinose. After 24 h of induction at 30 ℃ and 200 rpm, the cell cultures were harvested by centrifugation at 5,000 rpm for 5 min. The resulting cell pellets were resuspended in the reaction mixture (100 mM PBS, 50 mM glucose, 1 g/L l-histidine, 1 g/L l-methionine, 1 g/L L-cysteine, 20 mg/L FeSO_4_·7H_2_O, pH 7.0) to form a cell suspension (OD600 = 10). The whole-cell catalysis reaction was conducted in a 100-mL Erlenmeyer flask containing 30 mL of cell suspension on a rotary shaker (200 rpm) at 30 ℃ for 48 h. The extracellular and intracellular ERG content was subjected to high performance liquid chromatography (HPLC) analysis.

### Fed-batch cultivation

Precultures of the recombinant strains co-expressing *egt*1 and *egt*2 from *T. reesei* and *N. crassa* were prepared with ampicillin-containing LB medium in Erlenmeyer flasks at 37 °C at 200 rpm overnight. One hundred millilitres of the precultures were transferred into 900 mL of DM in 2-L jar fermenter, and cultivation was continued at 37 ℃, agitated with turbine impellers. When the OD_600_ of the cultures reached 30 (approximately 12 h), the inducer l-arabinose was added at a final concentration of 0.2%(w/v) to induce the expression of *egt*1 and *egt*2 at 30 ℃ with mixing for 12 h. After that, the amino acid mixture (40 g/L of each l-histidine, l-methionine and l-cysteine), which are the precursors of ERG biosynthesis, was constantly fed at a flow rate of 4 mL/h/L. During the whole fermentation, feeding solution (50% glucose, w/v) was periodically added after glucose depletion. The dissolved oxygen was kept above 20% air saturation by adjusting the agitation intensity and aeration rate. The pH was maintained at approximately 7.0 by automatic addition of 2.7 M ammonia solution or 1 M H_3_PO_4_. At the indicated time points, the cell cultures were sampled and used to detect extracellular ERG and *E. coli* growth as indicated by the optical density at 600 nm (OD_600_).

### HPLC analysis of ERG

ERG samples were diluted tenfold with a 70% acetonitrile solution. ERG standards (Std) were dissolved in a 70% acetonitrile solution. HPLC (Agilent 1200 infinity series 1260, Agilent Technologies) was performed with an Agilent ZORBAX NH_2_ column (4.6 × 250 mm, 5 μm). A mobile phase of acetonitrile/deionized water (70:30, v/v) was used at a flow rate of 1.0 mL/min. The produced ERG was detected at 254 nm and identified by comparison with the retention time of the analytical ERG standard (Sigma). Quantification was conducted by dividing the slope of the standard curves by the peak area.

### Liquid chromatography-mass spectrometry (LC–MS) analysis of ERG

HPLC-purified ERG was identified by LC–MS (Agilent 1260/6460LC/Triple Quadrupole MS, Agilent Technologies) with Agilent ZORBAX NH_2_ column (4.6 × 250 mm, 5 μm). Analysis was performed with a mobile phase of acetonitrile/4 mmol/L ammonium acetate (70:30, v/v).

## Supplementary Information

**Additional file1: Table S1** Primers used in this study. **Fig. S1 **LC-MS analysis of ERG. **A** ERG standards (10 ppm); **B** the ERG sample extracted from mycelia. **Fig. S2 **Plasmid profiles, protein expression and ERG production of BW-*ncget*1-*ncegt*2. **A** Schematic drawing of plasmids expressing Ncegt1 and Ncegt2 used in *E. coil* BW25113 transformation. **B** The production of ERG by 48-hour whole cell catalysis using the recombinant strains BW-*ncegt*1-*ncegt*2. Data in the figure are mean values (n = 3 biological replicates). **C** Detection of Ncegt1 and Ncegt2 expression in recombinant *E. coli* by SDS-PAGE. M. Protein marker; 1. BW-pBAD (control); 2. BW-*ncegt*1-*ncegt*2. **Data S1** Nucleotide sequences of* tregt*1 from *T. reesei* (2502 bp). **Data S2** Nucleotide sequences of* tregt*2 from *T. reesei* (1413 bp). **Data S3** Amino acid sequences of Tregt1 from *T. reesei* (833 aa). **Data S4** Amino acid sequences of Tregt2 from *T. reesei* (470 aa).

## Data Availability

All data generated or analyzed during this study are included in this published article and its additional files.

## Abbreviations

ERG: Ergothioneine
PDA: Potato dextrose agar
MM: Minimal medium
DM: Defined medium
HPLC: High performance liquid chromatography
LC–MS: Liquid chromatography mass spectrometry
