# Supplementary material for: Toward more efficient ergothioneine production using the fungal ergothioneine biosynthetic pathway
Source: Microb Cell Fact. 2022 May 7;21:76. doi: 10.1186/s12934-022-01807-3 (PMC9077841; doi:10.1186/s12934-022-01807-3)
Supplement: Supplementary file 1 — Additional file1: Table S1 Primers used in this study. Fig. S1 LC-MS analysis of ERG. A ERG standards (10 ppm); B the ERG sample extracted from mycelia. Fig. S2 Plasmid profiles, protein expression and ERG production of BW-ncget1-ncegt2. A Schematic drawing of plasmids expressing Ncegt1 and Ncegt2 used in E. coil BW25113 transformation. B The production of ERG by 48-hour whole cell catalysis using the recombinant strains BW-ncegt1-ncegt2. Data in the figure are mean values (n = 3 biological replicates). C Detection of Ncegt1 and Ncegt2 expression in recombinant E. coli by SDS-PAGE. M. Protein marker; 1. BW-pBAD (control); 2. BW-ncegt1-ncegt2. Data S1 Nucleotide sequences of tregt1 from T. reesei (2502 bp). Data S2 Nucleotide sequences of tregt2 from T. reesei (1413 bp). Data S3 Amino acid sequences of Tregt1 from T. reesei (833 aa). Data S4 Amino acid sequences of Tregt2 from T. reesei (470 aa). [file 12934_2022_1807_MOESM1_ESM.docx]

**Additional file 1**

**Table S1** Primers used in this study

| Primer name | Primers |
| --- | --- |
| FpBAD | GGCTGTTTTGGCGGATGAGA |
| RpBAD | GGTTAATTCCTCCTGTTAGCCC |
| Ftregt1 | GCTAACAGGAGGAATTAACCATGGCGCTCAAGTCAGCC |
| Rtregt1 | TCTCATCCGCCAAAACAGCCTCAAACATCTCGCACCAACCTT |
| Ftregt2 | GCTAACAGGAGGAATTAACCATGGCGTCTCTGCCGGTT |
| Rtregt2 | TCTCATCCGCCAAAACAGCCTCAAATCTTCTGGCCACCC |
| RpBAD-tregt1 | GGTATATCTCCTTCTTAAAGTTAAACATCAAACATCTCGCACCAACC |
| FpBAD-tregt2 | TTTAACTTTAAGAAGGAGATATACCATGGCGTCTCTGCCGG |
| FpBAD-ncegt1 | GCTAACAGGAGGAATTAACCATGCCGAGTGCCGAATCCATGAC |
| RpBAD-ncegt1 | CTCCTTCTTAAAGTTAAACATCACAAATCCCTAACAACTCTCGCCC |
| FpBAD-ncegt2 | TGTTTAACTTTAAGAAGGAGATATACCATGGTCGCCACCACCGTCGA |
| RpBAD-ncegt2 | TCTCATCCGCCAAAACAGCCTCAGGCGCTCTCCTTGTACTCC |


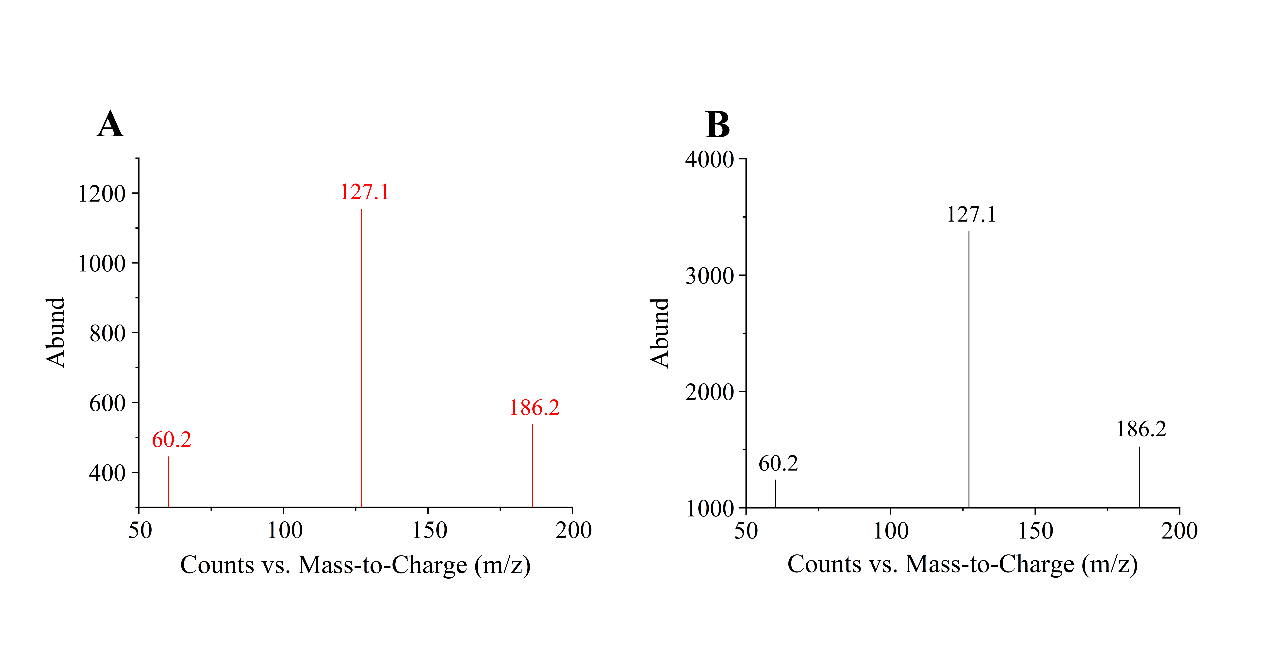


**Fig. S1 LC-MS analysis of ERG.** (A) ERG standards (10 ppm); (B) the ERG sample extracted from mycelia.


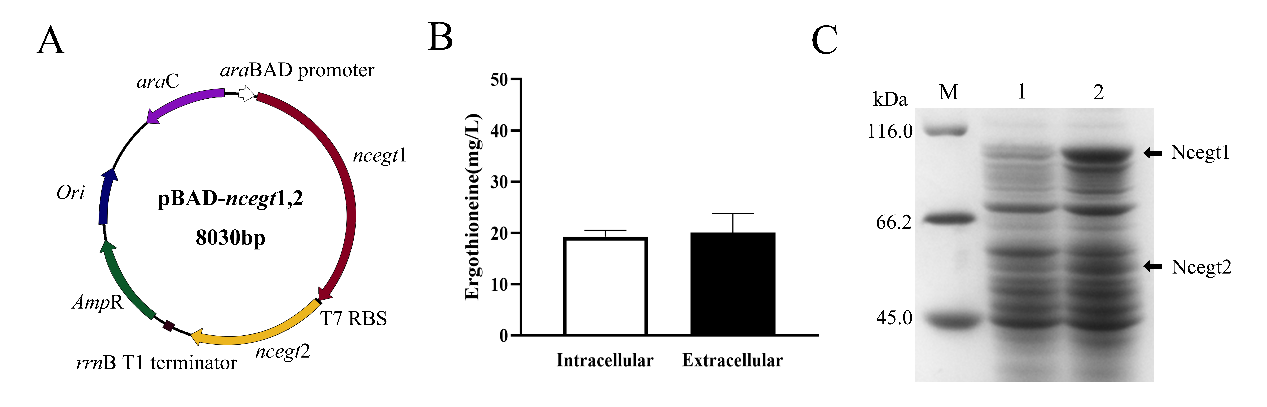


**Fig. S2 Plasmid profiles, protein expression and ERG production of BW-*ncget*1-*ncegt*2.** (A) Schematic drawing of plasmids expressing Ncegt1 and Ncegt2 used in *E. coil* BW25113 transformation. (B) The production of ERG by 48-hour whole cell catalysis using the recombinant strains BW-*ncegt*1-*ncegt*2. Data in the figure are mean values (n = 3 biological replicates). (C) Detection of Ncegt1 and Ncegt2 expression in recombinant *E. coli* by SDS–PAGE. M. Protein marker; 1. BW-pBAD (control); 2. BW-*ncegt*1-*ncegt*2.

**Data S1** Nucleotide sequences of *tregt*1 from *T. reesei* (2502 bp)

atggcgctca agtcagccac gaccacgact accaccacac agagggcgct cgacatcatc 60

gacattcaga atgcgcgaat cgaggtcaac ctcaaggacg agatccttgc ccagatgaat 120

cccgagcagg gcccccgcac gctgcccacg ctgctgctct atgacgagcg cggcctgcag 180

ctgtttgaag aaatcacgta tctggacgaa tactacctga ccaactacga gattgagctc 240

ctgaagaaat cggcagccga aatggccagc cagatccccg agggagcaat tgtggtggag 300

ctgggcagcg gaaacctgcg caaagtctgc ctcctcctgc aggcgtttga agatgccaag 360

aagaagattg actacttcgc gctcgacctg tcccagaagg agctcgagcg cacgctggcc 420

gaggcgcccg tctttgagta cgtgagctgc cggggcctgc gagggacgta tgacgacggg 480

tgcgagtggc tgaagcagga ggcgattctg gctcggccaa agtgcatttt gcacctcggc 540

tccagcattg gcaactttac gcgcgacgag gcggcagagt tcctgaggtc atttgcagag 600

gttctgcagc cctcggatct gatgattgtc ggggttgact catgtcagaa cccggacaag 660

gtgtaccatg catacaatga cagcaagggc gtcacacatc aattcgtcct caacggcctc 720

actcacgcaa acgaggttct cggtcaggaa gccttcaacg tcgaagagtg gcaagtcatt 780

ggcgaatacg tgtacgatgt cgacggcggc cgtcaccagg cctttgtgtc gcctctcgag 840

gtcgcctcgg tcctgggaca catcatcaag ccccacgagc gcatcaagat tgagcagagc 900

ttcaagtact ctgacgtcgg cctcgacaag ctgttcaaga cggccggcct cgtcgaggtt 960

gccaaatgga gccgccaggg cgaatacggc cttcacatgc tcaagagagc caagatgccc 1020

ttccccaggc tccgcgagct ttacgccagc gacaccctgc ccacctgggc cgactgggag 1080

aacctctggg cggcctggga cacggtcacc cgcaagatgc tccccgacgc cgagctcaac 1140

gagaagccca tcaagctgcg caacgcctgc atcttctacc tcggccacat cccggccttc 1200

ctcgacatcc agctcaagaa gacgaccaag gccggcggaa ccgagccgct ctacttccac 1260

accatctttg agcgcggcat cgacccggac gtcgacaacc cggaaaagtg ccacgaccac 1320

tccgaggtcc cggacgagtg gcctcccctt gaggacatcc tgaagtatca ggaccgcgtc 1380

cgcgagagac tccgcaagct gtatgccagc cccgatgagc ttgtcggaga cgttcggcgc 1440

gccgtctgga tcggcttcga gcacgaggcg cttcacctcg agacgctgct gtacatgctg 1500

ctgcagagcg acaagacgct tccgccgccg cacacggtgg tgccggactt ccccaagatg 1560

gcgcagaagg cgtatgccgc gcgggtgccg aatcagtggt ttgatgtccc ggagcagacg 1620

attaccattg gcatggacga tcctgaggat gagcacgaat caaaccggca ctttggatgg 1680

gacaacgaga agcctgccag acaggagact gtgcgtgcct ttcaggccaa ggccaggccc 1740

atcaccaacg aggagtacgc caagtacctc tactcttctc acattgagaa cctcccggcc 1800

tcttggtcgg tcatccctcc caactatcac cacaacacca acgccacgac gcccgggaag 1860

cccatcttga gcgagctccc cgagagcttc ctccacgaca aggcggtgcg gaccgtctac 1920

gggctggtgc ccctgcgcta cgccctcgac tggcctgtct ttgcgtcgta cgacgagctt 1980

gccggctgcg cggcgtggat gggcggcagg atcccgacga tggaggaggc caagagcatc 2040

tacgcgtatg tggagaggca aaaggatatt gccaagcaga gcaagctctc caacaaggtt 2100

ccagccgtca acgggcacct cgtcaatgac ggcgtcgaag agactccccc atccaagccc 2160

tccccggcct ccctcttcgt cgacctcagc acaaccaaca ccggcttcct ccactggcac 2220

cccgtccccg tgacccccaa cggcggctcc ctcgccggcc aggccgagct gggcggcgtc 2280

tgggagtgga cgagctccgt gctgcgtccg caccaggggt tccgcccgat gagcctctac 2340

ccgggctaca cggcggactt cttcgacgac aagcacaacg tcgtgctggg cgggtcctgg 2400

gcgacgcatc cccggattgc gggccggaag agctttgtca attggtatca gaggaattat 2460

ctgtatgcct gggttggggc aaggttggtg cgagatgttt ga 2502

**Data S2** Nucleotide sequences of *tregt*2 from *T. reesei* (1413 bp)

atggcgtctc tgccggttcg tcagcgagag gaaggagagg cgagggttgg cgaggatggc 60

ttcaaggtgt ttggcggcga gatgaagaag gactttttgt ttgctcccgg gtggacgaac 120

ctcaaccacg gctcgtacgg caccatcccc agggccatcc aagcaaaact gcgcagctac 180

caagacgaca ttgaggctcg tcccgacccc tttatccgct tcgagcacgc ccgcctgacg 240

gacgaatccc gcgccgccgt cgcgggcgtg ctcaacgtcc ccgtcgagac agtcgtcttc 300

gtcaacaacg cgaccgaggg cgtcaacacc gtcttccgca acatcaagtg ggacgccgac 360

ggcaaggacg tggcgctctg gttctcgacc gtgtacgagg cgtgcggcaa ggcgattgat 420

ttcctgtacg actaccacgg ggacggacgg ctgtcgagcc gggagattga gattgcgtat 480

ccgatcgagg acgacgagat cctgcggcgc ttccggagcg cggtggagca ggtccggagc 540

gaggggaagc gcgccaagat ttgcatcttt gacgtggtgt cgtcgcggcc gggcgtggtg 600

tttccctggg agcgcatggt ggctgcgtgt cgcgagctgg gcgtgctgag cctcgtggac 660

ggcgcgcagg ggatcggcat ggtgaggctg gatctcgggg ccgcggatcc ggatttcttc 720

gtgtcgaatt gtcacaagtg gttgtttacg ccgaggggtt gcgcggtgtt ttacgtgcct 780

gtgcggaatc agccgttgtt gccgtcgacg ctggcgacga gtcatgggta tgcttcgttg 840

acggggaaga ggagggcgcc ggcggggaag catgaagatg atgataatga tgacggttct 900

ttgaagaaga gcgcgtttgt gagcaacttt gagtttacgg ggacgaggga ctatacgccg 960

aatttctgtg tcaaggatgc ggttgcgtat cggagggatg tgctgggtgg ggaggagagg 1020

attttggagt atctgtggga tttgaataag aaggggagta ggcttgttgc ggagaggctg 1080

ggcacggagg tgttggagaa taaagagggg acgttgacga actgcgcgat ggcgaacatt 1140

gccatgcctc tgtggaaggg cgaggcaggc aaggaggggg atgttgttgt gcctgaggag 1200

gatggggatc gggtggttgt gtggatgatg agcacgatgg cgaaggatta caatacgatt 1260

gtgcccatgt tttggctcgg gaagaggttc tgggtgagga tcagtgcgca ggtgtatttg 1320

gatttgggag attatgagta tggcgcggag acgttgaaga agttgattga gagggttggc 1380

aagggggagt ataagggtgg ccagaagatt tga 1413

**Data S3** Amino acid sequences of Tregt1 from *T. reesei* (833 aa)

Met Ala Leu Lys Ser Ala Thr Thr Thr Thr Thr Thr Thr Gln Arg Ala

1 5 10 15

Leu Asp Ile Ile Asp Ile Gln Asn Ala Arg Ile Glu Val Asn Leu Lys

20 25 30

Asp Glu Ile Leu Ala Gln Met Asn Pro Glu Gln Gly Pro Arg Thr Leu

35 40 45

Pro Thr Leu Leu Leu Tyr Asp Glu Arg Gly Leu Gln Leu Phe Glu Glu

50 55 60

Ile Thr Tyr Leu Asp Glu Tyr Tyr Leu Thr Asn Tyr Glu Ile Glu Leu

65 70 75 80

Leu Lys Lys Ser Ala Ala Glu Met Ala Ser Gln Ile Pro Glu Gly Ala

85 90 95

Ile Val Val Glu Leu Gly Ser Gly Asn Leu Arg Lys Val Cys Leu Leu

100 105 110

Leu Gln Ala Phe Glu Asp Ala Lys Lys Lys Ile Asp Tyr Phe Ala Leu

115 120 125

Asp Leu Ser Gln Lys Glu Leu Glu Arg Thr Leu Ala Glu Ala Pro Val

130 135 140

Phe Glu Tyr Val Ser Cys Arg Gly Leu Arg Gly Thr Tyr Asp Asp Gly

145 150 155 160

Cys Glu Trp Leu Lys Gln Glu Ala Ile Leu Ala Arg Pro Lys Cys Ile

165 170 175

Leu His Leu Gly Ser Ser Ile Gly Asn Phe Thr Arg Asp Glu Ala Ala

180 185 190

Glu Phe Leu Arg Ser Phe Ala Glu Val Leu Gln Pro Ser Asp Leu Met

195 200 205

Ile Val Gly Val Asp Ser Cys Gln Asn Pro Asp Lys Val Tyr His Ala

210 215 220

Tyr Asn Asp Ser Lys Gly Val Thr His Gln Phe Val Leu Asn Gly Leu

225 230 235 240

Thr His Ala Asn Glu Val Leu Gly Gln Glu Ala Phe Asn Val Glu Glu

245 250 255

Trp Gln Val Ile Gly Glu Tyr Val Tyr Asp Val Asp Gly Gly Arg His

260 265 270

Gln Ala Phe Val Ser Pro Leu Glu Val Ala Ser Val Leu Gly His Ile

275 280 285

Ile Lys Pro His Glu Arg Ile Lys Ile Glu Gln Ser Phe Lys Tyr Ser

290 295 300

Asp Val Gly Leu Asp Lys Leu Phe Lys Thr Ala Gly Leu Val Glu Val

305 310 315 320

Ala Lys Trp Ser Arg Gln Gly Glu Tyr Gly Leu His Met Leu Lys Arg

325 330 335

Ala Lys Met Pro Phe Pro Arg Leu Arg Glu Leu Tyr Ala Ser Asp Thr

340 345 350

Leu Pro Thr Trp Ala Asp Trp Glu Asn Leu Trp Ala Ala Trp Asp Thr

355 360 365

Val Thr Arg Lys Met Leu Pro Asp Ala Glu Leu Asn Glu Lys Pro Ile

370 375 380

Lys Leu Arg Asn Ala Cys Ile Phe Tyr Leu Gly His Ile Pro Ala Phe

385 390 395 400

Leu Asp Ile Gln Leu Lys Lys Thr Thr Lys Ala Gly Gly Thr Glu Pro

405 410 415

Leu Tyr Phe His Thr Ile Phe Glu Arg Gly Ile Asp Pro Asp Val Asp

420 425 430

Asn Pro Glu Lys Cys His Asp His Ser Glu Val Pro Asp Glu Trp Pro

435 440 445

Pro Leu Glu Asp Ile Leu Lys Tyr Gln Asp Arg Val Arg Glu Arg Leu

450 455 460

Arg Lys Leu Tyr Ala Ser Pro Asp Glu Leu Val Gly Asp Val Arg Arg

465 470 475 480

Ala Val Trp Ile Gly Phe Glu His Glu Ala Leu His Leu Glu Thr Leu

485 490 495

Leu Tyr Met Leu Leu Gln Ser Asp Lys Thr Leu Pro Pro Pro His Thr

500 505 510

Val Val Pro Asp Phe Pro Lys Met Ala Gln Lys Ala Tyr Ala Ala Arg

515 520 525

Val Pro Asn Gln Trp Phe Asp Val Pro Glu Gln Thr Ile Thr Ile Gly

530 535 540

Met Asp Asp Pro Glu Asp Glu His Glu Ser Asn Arg His Phe Gly Trp

545 550 555 560

Asp Asn Glu Lys Pro Ala Arg Gln Glu Thr Val Arg Ala Phe Gln Ala

565 570 575

Lys Ala Arg Pro Ile Thr Asn Glu Glu Tyr Ala Lys Tyr Leu Tyr Ser

580 585 590

Ser His Ile Glu Asn Leu Pro Ala Ser Trp Ser Val Ile Pro Pro Asn

595 600 605

Tyr His His Asn Thr Asn Ala Thr Thr Pro Gly Lys Pro Ile Leu Ser

610 615 620

Glu Leu Pro Glu Ser Phe Leu His Asp Lys Ala Val Arg Thr Val Tyr

625 630 635 640

Gly Leu Val Pro Leu Arg Tyr Ala Leu Asp Trp Pro Val Phe Ala Ser

645 650 655

Tyr Asp Glu Leu Ala Gly Cys Ala Ala Trp Met Gly Gly Arg Ile Pro

660 665 670

Thr Met Glu Glu Ala Lys Ser Ile Tyr Ala Tyr Val Glu Arg Gln Lys

675 680 685

Asp Ile Ala Lys Gln Ser Lys Leu Ser Asn Lys Val Pro Ala Val Asn

690 695 700

Gly His Leu Val Asn Asp Gly Val Glu Glu Thr Pro Pro Ser Lys Pro

705 710 715 720

Ser Pro Ala Ser Leu Phe Val Asp Leu Ser Thr Thr Asn Thr Gly Phe

725 730 735

Leu His Trp His Pro Val Pro Val Thr Pro Asn Gly Gly Ser Leu Ala

740 745 750

Gly Gln Ala Glu Leu Gly Gly Val Trp Glu Trp Thr Ser Ser Val Leu

755 760 765

Arg Pro His Gln Gly Phe Arg Pro Met Ser Leu Tyr Pro Gly Tyr Thr

770 775 780

Ala Asp Phe Phe Asp Asp Lys His Asn Val Val Leu Gly Gly Ser Trp

785 790 795 800

Ala Thr His Pro Arg Ile Ala Gly Arg Lys Ser Phe Val Asn Trp Tyr

805 810 815

Gln Arg Asn Tyr Leu Tyr Ala Trp Val Gly Ala Arg Leu Val Arg Asp

820 825 830

Val

**Data S4** Amino acid sequences of Tregt2 from *T. reesei* (470 aa)

Met Ala Ser Leu Pro Val Arg Gln Arg Glu Glu Gly Glu Ala Arg Val

1 5 10 15

Gly Glu Asp Gly Phe Lys Val Phe Gly Gly Glu Met Lys Lys Asp Phe

20 25 30

Leu Phe Ala Pro Gly Trp Thr Asn Leu Asn His Gly Ser Tyr Gly Thr

35 40 45

Ile Pro Arg Ala Ile Gln Ala Lys Leu Arg Ser Tyr Gln Asp Asp Ile

50 55 60

Glu Ala Arg Pro Asp Pro Phe Ile Arg Phe Glu His Ala Arg Leu Thr

65 70 75 80

Asp Glu Ser Arg Ala Ala Val Ala Gly Val Leu Asn Val Pro Val Glu

85 90 95

Thr Val Val Phe Val Asn Asn Ala Thr Glu Gly Val Asn Thr Val Phe

100 105 110

Arg Asn Ile Lys Trp Asp Ala Asp Gly Lys Asp Val Ala Leu Trp Phe

115 120 125

Ser Thr Val Tyr Glu Ala Cys Gly Lys Ala Ile Asp Phe Leu Tyr Asp

130 135 140

Tyr His Gly Asp Gly Arg Leu Ser Ser Arg Glu Ile Glu Ile Ala Tyr

145 150 155 160

Pro Ile Glu Asp Asp Glu Ile Leu Arg Arg Phe Arg Ser Ala Val Glu

165 170 175

Gln Val Arg Ser Glu Gly Lys Arg Ala Lys Ile Cys Ile Phe Asp Val

180 185 190

Val Ser Ser Arg Pro Gly Val Val Phe Pro Trp Glu Arg Met Val Ala

195 200 205

Ala Cys Arg Glu Leu Gly Val Leu Ser Leu Val Asp Gly Ala Gln Gly

210 215 220

Ile Gly Met Val Arg Leu Asp Leu Gly Ala Ala Asp Pro Asp Phe Phe

225 230 235 240

Val Ser Asn Cys His Lys Trp Leu Phe Thr Pro Arg Gly Cys Ala Val

245 250 255

Phe Tyr Val Pro Val Arg Asn Gln Pro Leu Leu Pro Ser Thr Leu Ala

260 265 270

Thr Ser His Gly Tyr Ala Ser Leu Thr Gly Lys Arg Arg Ala Pro Ala

275 280 285

Gly Lys His Glu Asp Asp Asp Asn Asp Asp Gly Ser Leu Lys Lys Ser

290 295 300

Ala Phe Val Ser Asn Phe Glu Phe Thr Gly Thr Arg Asp Tyr Thr Pro

305 310 315 320

Asn Phe Cys Val Lys Asp Ala Val Ala Tyr Arg Arg Asp Val Leu Gly

325 330 335

Gly Glu Glu Arg Ile Leu Glu Tyr Leu Trp Asp Leu Asn Lys Lys Gly

340 345 350

Ser Arg Leu Val Ala Glu Arg Leu Gly Thr Glu Val Leu Glu Asn Lys

355 360 365

Glu Gly Thr Leu Thr Asn Cys Ala Met Ala Asn Ile Ala Met Pro Leu

370 375 380

Trp Lys Gly Glu Ala Gly Lys Glu Gly Asp Val Val Val Pro Glu Glu

385 390 395 400

Asp Gly Asp Arg Val Val Val Trp Met Met Ser Thr Met Ala Lys Asp

405 410 415

Tyr Asn Thr Ile Val Pro Met Phe Trp Leu Gly Lys Arg Phe Trp Val

420 425 430

Arg Ile Ser Ala Gln Val Tyr Leu Asp Leu Gly Asp Tyr Glu Tyr Gly

435 440 445

Ala Glu Thr Leu Lys Lys Leu Ile Glu Arg Val Gly Lys Gly Glu Tyr

450 455 460

Lys Gly Gly Gln Lys Ile

465 470
